# Comparative analysis of integrative classification methods for multi-omics data

**DOI:** 10.1093/bib/bbae331

**Published:** 2024-07-10

**Authors:** Alexei Novoloaca, Camilo Broc, Laurent Beloeil, Wen-Han Yu, Jérémie Becker

**Affiliations:** BIOASTER Research Institute, 40 avenue Tony Garnier, F-69007 Lyon, France; BIOASTER Research Institute, 40 avenue Tony Garnier, F-69007 Lyon, France; BIOASTER Research Institute, 40 avenue Tony Garnier, F-69007 Lyon, France; Bill & Melinda Gates Medical Research Institute, Cambridge, Massachusetts, MA 02139, United States; BIOASTER Research Institute, 40 avenue Tony Garnier, F-69007 Lyon, France

**Keywords:** benchmark, data integration, multi-omics data, prediction models, supervised analysis

## Abstract

Recent advances in sequencing, mass spectrometry, and cytometry technologies have enabled researchers to collect multiple ’omics data types from a single sample. These large datasets have led to a growing consensus that a holistic approach is needed to identify new candidate biomarkers and unveil mechanisms underlying disease etiology, a key to precision medicine. While many reviews and benchmarks have been conducted on unsupervised approaches, their supervised counterparts have received less attention in the literature and no gold standard has emerged yet. In this work, we present a thorough comparison of a selection of six methods, representative of the main families of intermediate integrative approaches (matrix factorization, multiple kernel methods, ensemble learning, and graph-based methods). As non-integrative control, random forest was performed on concatenated and separated data types. Methods were evaluated for classification performance on both simulated and real-world datasets, the latter being carefully selected to cover different medical applications (infectious diseases, oncology, and vaccines) and data modalities. A total of 15 simulation scenarios were designed from the real-world datasets to explore a large and realistic parameter space (*e.g.* sample size, dimensionality, class imbalance, effect size). On real data, the method comparison showed that integrative approaches performed better or equally well than their non-integrative counterpart. By contrast, DIABLO and the four random forest alternatives outperform the others across the majority of simulation scenarios. The strengths and limitations of these methods are discussed in detail as well as guidelines for future applications.

## Introduction

The continuous progress made in omic technologies have reshaped our understanding of human biology. Genome-wide association studies have, for example, successfully identified polygenic risk scores capturing genetic predisposition to complex diseases [1]. Similarly, transcriptomics studies have unveiled the molecular mechanisms underlying physiological (*e.g.* development stages, cell cycle phases) and pathological states, leading to clinical applications such as MammaPrint^®^, a 70-gene panel predicting the risk of relapse and metastasis in breast cancer [2]. While single omic analyses have produced valuable insights, the majority of common human diseases associated with high mortality (*e.g.* type 2 diabetes, cardiovascular disease) still lack effective therapeutic strategies though [3]. For instance, the functions underlying genetic variants are not easily inferred and have failed to provide targeted treatments. These observations have led to a growing consensus that a holistic approach is needed to identify new candidate biomarkers and unveil mechanisms underlying disease etiology, both key to precision medicine.

The past decade has witnessed an increased number of large-scale cancer projects (TCGA [4], ICGC [5], COSMIC [6], TARGET [7], DKTK [8]) that consistently demonstrated the power of data integration in patients stratification. In a lung adenocarcinoma study for example, Gillette *et al.* [9] proposed a refined classification by dividing the proximal-proliferative cluster using transcriptomic, deep-scale proteomic, and post-translational modifications.

Due to the relative novelty of the field, numerous challenges remain in integrative analysis such as (i) high dimensionality that significantly impacts inference; (ii) data heterogeneity inherent to heterogeneous technical sources of variability across platforms, thereby reducing the biological signal; (iii) the diversity of data types making a ”one method fits all omics” unlikely to exist; and (iv) interpretation, where the huge amount of information makes meaningful conclusions difficult to draw.

In this context, a wide variety of integrative approaches have been introduced to address one or some of the following goals: (i) patient stratification, (ii) prediction of clinical outcome, and (iii) identification of molecular mechanisms acting across molecular layers. In this regard, recent works applied causal inference to either test known biological relationships [10] or infer stable relations across multiple experimental conditions, without prior knowledge [11]. Different classifications have been proposed based on the application (unsupervised, supervised, the latter being further subdivided into predictive and explanatory), strategy (early, intermediate, late), and underlying methodology. The current literature commonly distinguishes six families of integrative methods: matrix factorization, Bayesian, multiple kernel learning, ensemble learning, deep learning, and network-based methods [12–19].

While a broad spectrum of unsupervised integrative methods have been developed and extensively reviewed [20–24], their supervised counterparts have received less attention in the literature. At the time of writing, a handful of studies were found to cover the topic. Cai *et al.* [25] and Cantini *et al.* [18] evaluated dimension reduction techniques both on supervised and unsupervised aspects. The first demonstrated that drug response was best predicted by early integration (concatenation) or moCluster, a matrix factorization method, both combined with random forest (RF). The second showed that multiple co-inertia analysis achieved the best performance on cancer survival prediction. Still on cancer survival analysis, Herrmann *et al.* [26] compared 11 boosting, RF and penalized regression approaches on TCGA and concluded that methods taking into account the multi-omics structure (*i.e.* the per omic variable grouping) outperformed the Cox model by a small margin, trained on clinical variables alone. Wissel *et al.* [27] confirmed this result on TCGA, but observed an opposite trend on ICGC and TARGET datasets where multi-omics data led to improved performance relative to clinical-only models. They further highlighted the superiority of penalized Cox model and random survival forest over neural networks in terms of model calibration. Leng *et al.* [28] compared 15 deep learning methods on simulated, single-cell and cancer multi-omics datasets. Among the six supervised models evaluated on categorical responses, a graph attention network method, moGAT, consistently achieved higher performances both on simulated and experimental datasets.

With a growing interest in extracting multi-omics features associated with health-related outcomes, an in-depth understanding of current supervised integrative approaches is much needed. Since little has been done outside survival analysis, we propose in this work to evaluate six supervised methods, representative of the main families of intermediate integrative approaches (Table 1), in a classification setting. The methods were selected based on five criteria: code availability, applicability to any data type, statistical validity, ability to perform variable selection, and ability to account for prior knowledge. Although the methods were evaluated solely on a predictive criterion (see Methods section), their selection was also guided by their interpretability capabilities, since feature prioritization often suffers from poor alignment with known molecular mechanisms. For this reason, two methods incorporate a priori biological knowledge into their model. As non-integrative control, RF was additionally performed on each data type separately.

**Table 1:** Summary of the methods selected in the benchmark. Prior information indicates whether the method takes prior biological knowledge into account. Package version are indicated within brackets.

Approach	Name	Underlying model	Prior information	Implementation
Integrative	DIABLO	Sparse generalized CCA	No	R package *mixOmics* (6.16.3)
	SIDA	Combination of LDA and CCA	Yes	R package *SIDA* (1.0)
	PIMKL	Multiple kernel learning	Yes	Python script (0.1.1)
	netDx	Integrated patient Similarity network	Yes	R package *netDx* (1.4.3)
	Stacked generalization	Ensemble of weak learners	No	R package *SuperLearner* (2.0-28)
	Block Forest		No	R package *BlockForest* (0.2.6)
Non-integrative	RF_Concat RF_Max_Single_View	RF on concatenated data separated data	No	R package *randomForest* (4.7-1)

**Figure 1:**
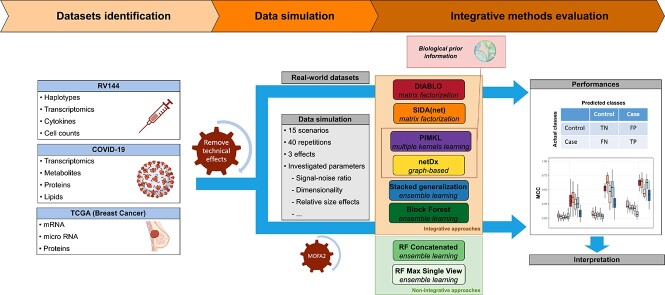
Schematic of the benchmark workflow. Three multi-omics datasets, covering distinct medical applications, were selected. A reference simulation scenario was designed using SNR and sparsity levels estimated from real-world datasets. A total of 14 alternatives were also generated by modifying class imbalance, SNR, dimensionality, relative importance of effects, etc. A selection of six integrative approaches, representative of existing methods, were evaluated on both real-world and simulated data based using MCC.

Methods were evaluated on both simulated and real-world datasets, the latter being carefully selected to cover different medical applications (infectious diseases, oncology, and vaccine) and data modalities (Fig. 1). A set of 16 simulations were designed from the real-world datasets to explore a large and realistic parameter space (e.g. sample size, dimensionality, confounding effects, effect size).

The remainder of the paper is organized as follows: the next section briefly introduces the methods, simulation scenarios, multi-omics datasets, and evaluation criteria used in this study. The results section presents the relative performances of the methods on simulated and experimental data. Finally, in the light of the results, we discuss the strengths and limits of these methods and provide guidelines for future applications. In the rest of the paper, the terms data type, modality, and view are used interchangeably.

## Methods

### Methods overview

Consider a K-class classification problem with $Q$ matrices $X_{q}$ of dimensions $N \times p_{q}$, $(q = 1, \cdots , Q)$ measured on the same $N$ samples.

Data Integration Analysis for Biomarker discovery using Latent cOmponents (**DIABLO**) seeks shared variations across data types while simultaneously discriminating phenotypic groups [29]. DIABLO extends sparse generalized canonical correlation analysis (sGCCA) to a supervised framework by substituting one view with a vector of outcome. sGCCA builds linear combinations that maximize the sum of pairwise covariance across modalities [30]. DIABLO solves a similar optimization function for each component $h \in [1, \dots , H]$: 


(1) \begin{align*}& \begin{aligned} \max_{a_{h}^{(1)}, \dots, a_{h}^{(Q)}} \sum_{i,j=1, i \neq j}^{D} c_{i,j} \; \text{cov}\left(X_{h}^{(i)} a_{h}^{(i)}, X_{h}^{(j)} a_{h}^{(j)}\right) \\ \text{s.t.} \; ||a_{h}^{(q)}||_{2} = 1 \; \text{and} \; ||a_{h}^{(q)}||_{1} = \lambda^{(q)} \; \text{with} \; 1 \le q \le Q \end{aligned}\end{align*}

where $X_{h}^{(i)}$ is the depleted matrix after iteration $h-1$, $A^{(i)} = [a_{1}^{(i)}, \dots , a_{H}^{(i)}]$ the loading matrix in view $i \in [1, \dots , Q]$, and $c_{i,j}$ an element of the design matrix $C$ specifying whether views $i$ and $j$ are connected. This information is commonly provided by the user; alternatively, connections can be learnt from the data, using a threshold on the correlation between the first component of each omics. A $\ell _{1}$ penalization is applied on the coefficients of the linear combinations to select variables that are most correlated within and between modalities. In a predictive perspective, the number of components and variables to select is determined by minimizing the cross-validation error. In a similar way to linear discriminant analysis (LDA), K-1 components are sufficient to discriminate K classes. Alternatively, an in-depth biological interpretation requires a larger set of variables to perform gene set enrichment analysis. The method classifies new samples based on their similarity in the latent space with classes in the training set using a predefined distance. The predictions are generated at the view level and then combined through a majority vote. This majority vote is weighted by the correlation between the latent components and the outcome on the training set. In this way, DIABLO can discard views that are not informative.

Sparse Integrative Discriminant Analysis (**SIDA**) approaches integration as a joint separation and association problem by combining LDA and canonical correlation analysis (CCA) [31]. LDA seeks linear combinations such that sample projections have maximal separation between classes and minimal separation within classes. CCA on the other hand finds linear combinations in each modality in a way that their pairwise correlation is maximized. In a $K$-class classification problem with $D = 2$, SIDA seeks $(K-1)$ eigenvectors $A=[ \alpha _{1}, \dots , \alpha _{(K-1)} ] \in \mathrm{I\!R}^{p_{1} \times (K-1)}$ and $B=[ \beta _{1}, \dots , \beta _{(K-1)} ] \in \mathrm{I\!R}^{p_{2} \times (K-1)}$ associated with $X_{1}$ and $X_{2}$ that maximize the objective function:


(2) \begin{align*}& \begin{aligned} \max_{A,B} \rho \, \text{tr} \left( A^{T}S^{b}_{1}A+ B^{T}S^{b}_{2}B \right) + \left( 1- \rho \right) \text{tr} \left( A^{T}S_{12}BB^{T} S_{12}^{T}A \right) \\ \text{s.t.} \; \text{tr}(A^{T}S_{1}^{w} A)/(K-1)= (B^{T}S_{2}^{w} B)/(K-1) = 1 \end{aligned}\end{align*}

where tr is the trace function, $\rho $ the parameter controlling the relative importance of LDA and CCA (set by default at 0.5), $S_{q}^{b}$ and $S_{q}^{w}$ the between and within classes covariances in dataset $q$, and $S_{12}$ the cross covariance between views. The eigenvectors are estimated using Lagrangian multipliers.

The algorithm also performs variable selection by applying a block $\ell _{1}/\ell _{2}$ penalty on the eigenvectors. Another specificity of SIDA is the possibility to include adjustment covariates in the model to guide the selection of relevant variables likely to improve classification accuracy. SIDA extension, SIDANet, can incorporate prior knowledge in the form of a network. This prior information is again included in the penalty function applied on the eigenvectors. Finally, similar to DIABLO, new samples are classified based on their similarities between their coordinates in the latent space and classes in the training set. The main difference lies in that SIDA computes similarities on all latent variables concatenated across views.

Pathway Induced Multiple Kernel Learning (**PIMKL**) computes kernels on separate feature sets (biological pathways) and linearly combines them such that the resulting kernel correlates with a response variable (see Equation 5). The combination of data types through multiple kernels aims to both increase the predictive power and facilitate the interpretability of the model, since weights reflect the importance of feature sets on the classification problem. The method relies on the concept of pathway induction that consists in building kernels using both an interaction network and a pathway database. In case such prior knowledge is not available, kernels can alternatively be built on the full datasets. Let $X^{p}_{q}$ be the submatrix of $X_{q}$ restricted to the features in pathway $p$. Each pathway-induced kernel is built using a Gram matrix $K^{p}$ defined as follows:


(3) \begin{align*}& K^{p} = {X_{d}^{p}}^{T} \mathcal{L}^{p} X_{d}^{p}\end{align*}

For a pair of samples $i,j \in{1,\dots , N}$, the entries of the Laplacian matrix $\mathcal{L}^{p}$ is defined as


(4) \begin{align*}& \begin{aligned} \text{ with} \mathcal{L}^{p}_{ij} =\left\{\begin{matrix} 1 - \frac{\text{w}(i,j)}{d_{i}} & \text{if\ } i = j \text{ and} d_{i} \neq 0 \\ \frac{\text{w}(i,j)}{\sqrt{d_{i} d_{j}}} & \text{if\ } i,j \text{ are adjacent} \\ 0 & \text{otherwise} \end{matrix}\right. \end{aligned},\end{align*}

where $d_{i}$ is the degree of node i in the graph and $\text{w}(i,j)$ the weight of $(i,j)$ in the interaction network. Kernels are then linearly combined using EasyMKL [32]: 


(5) \begin{align*}& K = \sum_{k=1}^{K} w_{p} K^{p},\end{align*}

where $w_{p}$ are the weights to optimize.

**netDx** is a classification framework that aims to predict patient clinical outcome using similarity network fusion [33]. In a similar way to PIMKL, (i) the method generates patient similarity networks (PSNs) on each data types and subsequently combine them into an integrated network; (ii) the method can either build PSN on data types as a whole or on subsets of features, and (iii) netDx provides biologically interpretable results. In a PSN, nodes correspond to samples and edges to pairwise similarity, calculated by default with a Pearson correlation (other built-in similarity measures are also available e.g. normalized or Euclidean distance). To improve method accuracy, netDx performs feature selection both at the variable and network levels using Lasso and a score measuring class homogeneity in each PSN. In the fusion step, the selected PSNs are combined by averaging their similarity scores to produce the final integrated network. New samples can then be classified using label propagation [34].

**Stacked Generalization**, also called stacking or super-learning, is an ensemble technique that combines multiple predictors trained on a single dataset to increase the predictive power [35]. Van der Laan *et al.* [36] demonstrated that when the number of samples is large, a super-learner performs at least as well as the best individual predictor. Predictors are commonly fused through a weighted sum named “convex combination,” motivated by theoretical results and improved stability [35]. While originally designed as a multiple classifier on a single dataset, it was recently extended to multi-omics by applying a single classifier, Elastic Net, independently on each modality [37]. This alternative means that the theory established on the initial version may no longer be valid. In contrast to Ghaemi *et al.* [38], we selected RF in this work due to its demonstrated accuracy in classification problems.

**BlockForest** is an extension of RF to multi-omics analysis that includes the group structure in the selection of the split points [39]. In standard RF, trees are grown by recursively dividing samples in two subgroups using the best split (in terms of Gini impurity) from a subset of randomly selected features. The specificity of BlockForest lies in that, at each node, both modalities and features are sampled with weights $b_{d}$, estimated from the data and $\sqrt{p_{d}}$, respectively.

As non-integrative control, the same classifier (RF) was also included in this benchmark to evaluate the added value of data integration. Two alternatives were considered, RF on concatenated or separated data types (**RF_Concat**, **RF_Max_Single_View**). The first concatenates omics layers sample-wise and evaluates the overall performance. The second consists in evaluating RF on each modality and keeping only the highest classification performance. A brief description of the methods, their implementation, and ability to account for prior information is available in Table 1.

### Simulation scenarios

The simulations were generated using MOFA, a linear latent variable model that decomposes data modalities into a matrix of shared factors ($\mathbf{Z}$) and Q weight matrices ($\mathbf{W^{1}},\cdots ,\mathbf{W^{Q}}$) [40]. Let $\mathbf{w^{q}_{k}}$ be the $k$-th column of $\mathbf{W^{q}}$ associated with factor $k$. The latent factors capture the main sources of variability on which downstream interpretation (*e.g.* clustering, pathway enrichment) can subsequently be carried out. Among the three likelihood models available, only the Gaussian noise was considered here. Weights were simulated from a product of two random variables, Gaussian and Bernoulli distributed. Factors, on the other hand, were simulated from a mixture of two Gaussian distributions centered on 0, with distinct precision parameters. For more details on the model, we refer the reader to the Supplementary Methods of Argelaguet *et al.* [40].

Although confounding factors are commonly adjusted for prior to data integration, other sources of variation (*e.g.* technical, environmental, demographic factors) may remain uncorrected in omics analyses [41]. Therefore, to best recapitulate experimental multi-omics data, three effects were simulated: a main multi-omics factor (Main_MO) and two confounding factors acting at the single- and multi-omics levels (Conf_SO, Conf_MO). This translates into the Factor matrix $\mathbf{Z}$ having three latent components, two that are shared and one that is omic-specific. While the first was designed to evaluate the methods’ ability to detect a shared signal across data types, the last two were introduced to assess their robustness against confounders.

A reference scenario was first devised using signal-to-noise ratio (SNR) and sparsity levels estimated by MOFA on the real-world datasets, the overall SNR being determined by the precision parameters of factors, weights and noise. Each simulation consists of $Q=3$ views $X_{q}$ of dimension $p_{q} \times N$, with N = 80 samples and $p_{q}$ features, with $\mathbf{p} = (1000, 240, 60)$, see Table 2. Although the dimensions are one order of magnitude smaller than typically measured in omics experiments, it is common practice to perform data integration on the most variable features, *i.e.* with the highest variance [42]. The three factors were sampled independently using the same Gaussian mixture with mixture coefficient $\pi =0.5$ and precision parameters $\tau = (0.1,1)$, resulting in orthogonal effects with identical magnitudes. The sparsity level of factor $k$ in view $q$ is expressed as the fraction of features with non-null weights in $\mathbf{w^{q}_{k}}$ and was set to $\theta =0.1$. The non-null weights were sampled using a Gaussian distribution with precision $\alpha = 0.1$. Among the features with non-null weight, referred to hereafter as “signal features,” 50% are shared with at least another factors. The remaining features, not driven by latent factors, consist of noise only.

**Table 2:** A total of 15 scenarios were generated from real-world datasets. The **reference** scenario is defined by two classes of 40 samples each, three omics with $p = (1000, 240, 60)$ variables and 3 factors, a main multi-omics (Main_MO), and two confounding factors acting at the single-omic (SO) and multi-omics (MO) levels, named hereafter Conf_SO, Conf_MO. The factors have same SNR, drive 10% of the features each, among which 50% are shared with at least one other factor. For the other scenarios, only the deviations from the reference are indicated. The first nine are shown in Fig. 2, the others in Supplementary Figure 1.

Scenario	Number of samples (cases, controls)	Number of features per omic	Main factor(s)	Fraction of signal features per omic	Overlap across factors
**Reference**	**80 (40, 40)**	**1000, 240, 60**	**All equal**	**0.1, 0.1, 0.1**	**0.5**
n/5	16 (8, 8)				
px5		5000, 1200, 300			
CaseControl_1:7	80 (10, 70)				
High_Main_MO			High main MO		
High_Conf_MO_Overlap			High confounding MO		0.95
Main_MO_2Smallest_Omics			Main MO kept in the		
Main_MO_1Largest_Omic			smallest/largest omic(s)		
High_Fraction_Signal_Feat				0.3, 0.3, 0.3	
nx5	400 (200, 200)				
p/5		200, 48, 12			
High_Conf_SO_Overlap			High confounding SO		0.95
Main_MO_1Smallest_Omic			Main MO kept in the		
Main_MO_2Largest_Omics			smallest/largest omic(s)		
Noise				0, 0, 0	

From this reference scenario, 14 alternatives were generated by modifying one or two parameters at a time. The influence of $n/p$ ratio was investigated in four scenarios where the number of samples or features was multiplied or divided by 5. The case to control ratio was also shifted from 1:1 to 1:7 in the main_MO factor to study the effect of class imbalance. To investigate the impact of confounders, the magnitude of each factor was raised separately by setting the precision of the Gaussian mixture to $\tau = (0.01,1)$. For the two confounding factors, the effect change was combined with an increased overlap across factors from 50% to 95%. Because multi-omics effects can sometime occur in a subset of views, the main_MO effect was simulated in the largest(s) or smallest(s) views only while keeping the other two effects unchanged. To investigate the influence of signal features, their fraction was raised to $\theta =0.3$. Finally, methods specificity was studied by setting the number of signal features to 0, leading to random noise matrices. For each scenario, 40 repetitions were generated. The parameters used in each scenario are summarized in Table 2. The code used here was derived from the make_example_data function from the MOFA2 R package.

### Real-world datasets

Since the SNR varies across medical applications, integrative methods were evaluated on real-world datasets derived from three distinct applications: vaccine (RV144 HIV), infectious disease (COVID-19), and oncology (TCGA breast cancer). For all three datasets, the same preprocessing steps were applied prior to data integration: (i) patients with missing clinical data and features with missing values were discarded, (ii) potential confounders were adjusted for, and (iii) only the 1000 most variable features were retained in large data types, leading to dimensions similar to those in the simulation study.

The first dataset is a case-control study by Fourati *et al.* [43] who characterized the molecular mechanisms underlying RV144 vaccine protection using a multi-omics approaches. The transcriptomic data revealed that IFN$\gamma $ stimulated genes are associated with a reduced risk of HIV-1 acquisition. In addition to the 47 323 transcripts, six cell types, nine cytokines, and 31 MHC class II alleles were measured. Although the number of variables differs by two orders of magnitude after dimension reduction, this dataset is representative of many vaccine studies. After data filtering, a total of 140 vaccinees and 21 placebo recipients were kept. Normalized data were adjusted for five clinical variables (age, sex, behavior risk, vaccination site, and enrollment date).

The second dataset consists of 102 COVID-19 samples stratified into high and low severity using a composite score that accounts for mortality and hospitalization duration [44]. A total of 517 proteins, 646 lipids, and 150 metabolites were identified by mass spectrometry in plasma and 13 263 transcripts in leukocytes by RNA-sequencing. The authors found 219 features strongly correlated with COVID-19 status and severity, pointing toward the modulation of several pathways, including lipid transport and neutrophil degranulation. Omic measurements were corrected for age, gender, ethnicity, and two samples were filtered out, leading to 49 severe and 51 less severe samples.

The TCGA breast cancer dataset was repeatedly used in the evaluation of integrative approaches and therefore also selected in this work [45–49]. This dataset consists of 90 489 SNPs, 20 531 mRNAs, 1046 miRNAs, 27 578 DNA methylations, and 226 proteins (reverse phase protein array) measured in 825 patients [50]. Among the eight histological types available in the clinical data, only the two most numerous (infiltrating ductal and lobular carcinoma) were retained to match the binary outcomes in the simulations and the other two real-word datasets. Since the five omics were not systematically measured in all patients, the number of patients in four omics was maximized by excluding the methylation dataset. The SNP dataset was also removed from the analysis, because some of the selected methods do not support binary data. The data filtering step led to a selection of 527 samples, 396 and 131 infiltrating ductal and lobular carcinoma, respectively. Three clinical variables, age, gender, and ethnicity, were adjusted for.

### Classification performance criteria

The performances were measured using two metrics commonly used with binary classifiers, the Matthews Correlation Coefficient (MCC) and $F_{1}$-score, defined as


(6) \begin{align*} MCC &= \frac{ TP \times TN - FP \times FN} {\sqrt{(TP+FP)(TP+FN)(TN+FP)(TN+FN)}} \end{align*}

(7) \begin{align*} F_{1} &= \frac{2 \times TP}{2 \times TP + FP + FN}, \end{align*}

where TP and TN are the number of true positives and negatives; and FP and FN the number of false positives and negatives. In a recent study, Chicco *et al.* [51] advocated for the use of MCC over accuracy and F1-score due to its robustness in imbalanced settings and invariance for class swapping [51]. For this reason, the performances are presented in the main text using MCC and are additionally provided as F1-scores in the Supplementary Figures 2–4. Like Pearson’s correlation coefficient, MCC ranges between -1 and 1. $\pm 1$ reflects perfect (mis)classification, while 0 indicates random classification. To ensure an unbiased comparison, all methods were evaluated in five-fold cross-validation.

### Method parametrization

Since K-1 dimensions are sufficient to discriminate K groups, the number of components was set to 1 in DIABLO and SIDA. According to the authors’ guidelines, DIABLO was run with a null design (views are only connected to the response variable) to maximize discrimination over cross-omic correlation. A Mahalanobis distance was applied to generate predictions at the view level that were subsequently aggregated using a weighted majority vote. Similar to DIABLO, the weight between LDA and CCA was set to 1 in SIDA to favor separation over association. Of note, the default range controlling variable selection in SIDA was expanded leading to a substantial increase in classification performance (details are provided in Supplementary Materials). In PIMKL and netDx, similarity networks and kernels can be built on full datasets or feature sets. Since this study is focused on performance prediction and that the other methods work at the dataset level, the former was preferred. In PIMKL, all features within a modality were consequently connected pairwise in the interaction network. The regularization parameter was left at its default value of 0.5. As suggested by the authors of netDx, a LASSO pre-filtering step was performed to select the most predictive features. In RF, the number of variables selected at each split and the minimum leaf size were tuned, as recommended by Hastie *et al.* [52]. Hyper-parameters were tuned on each repetition using nested cross-validation (sparsity level in DIABLO and SIDA) or cross-validation combined with out-of-bag error (number of variables to sample and leaf size in RF). In the case of stacked generalization, a two level nested cross-validation combined with out-of-bag error was used to tune RF hyper-parameters and generate predictions at the base and meta classifier levels. Of note, both DIABLO’s tuning and performance built-in functions run cross-validation on the full dataset. Given that this recommended workflow likely leads to inflated performance, nested cross-validation was instead preferred. The *Caret* R package was used to create balanced folds.

## Results

### Performances on simulations

**Figure 2:**
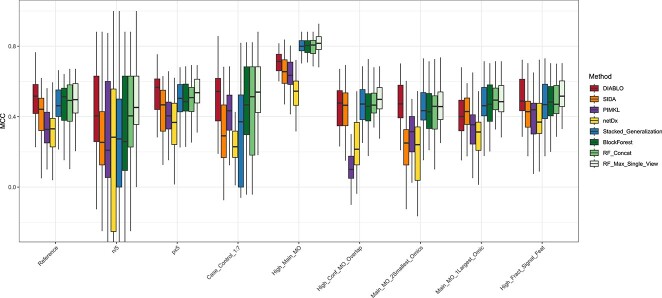
**Method comparison on simulated data.** Integrative approaches were evaluated on 15 simulation scenarios (nine displayed here, the others in Supplementary Figure 1). Two non-integrative methods (RF_Concat, RF_Max_Single_View) were also included to quantify the added-value of data integration. For each scenario, 40 repetitions were generated, on which MCC was computed in five-fold cross-validation.

In the reference scenario (Fig. 2), different behavior can be observed across methods. The median MCC ranges from 0.33 in netDx and PIMKL to 0.52 in DIABLO. Following closely are the four RF-based methods whose median MCC varies between 0.46 (Stacked Generalization) and 0.51 (BlockForest). SIDA comes just after Stacked Generalization with median MCC=0.44. When $n/5$, a sharp performance decrease can be noted for all methods but two (netDx and RF_Max_Single_View), along with an increased interquartile range. BlockForest, Stacked Generalization, and SIDA appear to be the most sensitive to small sample size with a $\approx $ 0.2 drop in median MCC. Conversely, in the $n\times 5$ scenario, the dispersion is decreased, with PIMKL and netDx reducing the gap with a median MCC$\approx $0.40 (see Supplementary Figure 1). These observations indicate that the number of samples has a major impact on performances and that all methods seem to converge toward a common MCC when $n$ is large enough.

By contrast, the number of features has a limited effect on performances, except for PIMKL whose accuracy seem to reflect dimensionality in $p\times 5$ and $p/5$ scenarios. However, three scenarios indicate that these variations can be attributed to the absence of feature selection. When the fraction of signal features (High_Fract_Signal_Feat) is raised, PIMKL shows the highest performance increase. Conversely, in High_Conf_[MS]O_Overlap, where confounding factors are increased and features are shared across factors, PIMKL displays degraded performance due to the absence of feature selection in kernels. Although less pronounced due to the LASSO pre-filtering step, a comparable accuracy reduction can be noticed with netDx. These observations are not unexpected since the two methods were originally designed to compute sample similarities on gene-sets, *i.e.* correlated variables. By contrast, latent variable models and RF-based methods maintain a high MCC on High_Conf_[MS]O_Overlap, reflecting their ability to select discriminant variables among a majority of noise and confounding features.

Despite having a similar underlying model, DIABLO and SIDA present two distinct behaviors when the main effect is missing in at least one view. When the main effect is present only in one or two of the smallest views, DIABLO’s median MCC remains nearly unchanged compared with the reference, indicating high robustness in this setting. Conversely, SIDA’s median MCC drops by 0.09 and 0.19 on the same comparison. This result is counter-intuitive as one would expect performance to rise with the number of views containing signal. In Main_MO_[12]largest_Omic on the other hand, no performance reduction is observed, suggesting that SIDA’s accuracy mainly depends on the largest views. Still on Main_MO_1largest_Omic, DIABLO’s median MCC decreases by 0.12. This change remains unexpected considering that DIABLO’s majority vote should down-weight views without main effect and therefore be robust to the absence of signal. The estimated weights show in fact the opposite: views without main effect tend to have larger contributions and more variables selected (Supplementary Figure 5). A good illustration is provided by the noise scenario where the median weight is 0.73 in the largest view. This paradoxical result arises from the fact that correlations between latent components and outcome are computed on the training set rather than the test set, explaining the observed inflated correlation values. The large number of selected variables is further indicative of the model’s inability to find predictive features.

When the case to control ratio is set to 1:7, PIMKL and the two non-integrative approaches exhibit an improved MCC, while SIDA, netDx, and stacked generalization performances are reduced by approximately 0.1. Looking more closely at the methods output, it appears that all methods but netDx predict the majority class almost systematically, reflecting a low discriminatory power in such imbalance setting. In High_Main_MO, where the main multi-omics factor has a larger SNR than the two confounders, the four RF-based methods reach a median MCC=0.8, *i.e.* 0.1–0.2 higher than the other four. This result suggests that when the main multi-omics effect is strong, efficient machine learning techniques can potentially overcome integrative approaches. On the same scenario, PIMKL narrows the gap with DIABLO and SIDA. Lastly, when the three views only consist of noise, the median MCC is comprised between -0.08 (SIDA) and 0.13 (RF_Max_Single_View, see Supplementary Figure 1). Although this range corresponds to random classifiers, it can however be noted that RF_Max_Single_View stands out, with the highest median MCC. This result was expected due to the post-hoc selection of the best performing modality.

To summarize the results on simulated data, (i) DIABLO achieved a high level of accuracy in most scenarios due to an efficient variable selection. The method however returned unreliable weights and lower performances in the Main_MO_1Largest_Omic scenario. (ii) SIDA demonstrated improved abilities to recover multi-omics signal in the high confounding, high overlapping setting. However, the method proved to be sensitive to small sample size, class imbalance, and to the absence of multi-omics effect in the largest modality. (iii) Because PIMKL was initially designed to build kernels on homogeneous sets of variables, performances were degraded when the fraction of signal variable was small. For the same reasons, PIMKL showed the highest performance increase when the signal features was raised to 30%. Although less pronounced, a similar trend was observed with netDx, whose underlying model is also based on sample similarities. (v) In most scenarios, Stacked Generalization showed average performances, with lower MCC than the other two RF-based approaches. This point is unexpected given that RF_Max_Single_View is a special case of Stacked Generalization when all the weight is put on one view. (vi) On average, RF_Concat and RF_Max_Single_View performed equally well as the best integrative approaches and even outperformed them when the main multi-omics effect was high, though they did not handle imbalanced designs well. At last, the CaseControl_1:7 scenario revealed that many methods were often biased toward the majority class in imbalanced settings. The runtimes are provided in Supplementary Table 1.

### Performances on real-world data

**Figure 3:**
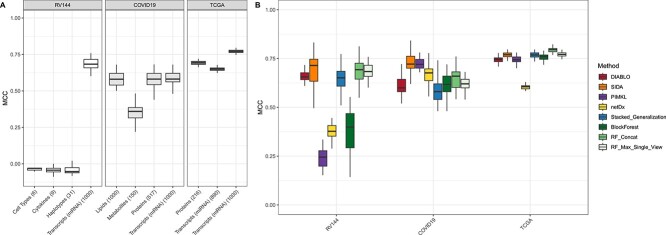
**Method comparison on three real-world datasets.** Prediction performance **(A)** on individual omic using RF or **(B)** integrative methods. MCC was computed on 40 repetitions of five-fold cross-validation.

The integrative methods were further evaluated on three real-world datasets. In addition to the integrative analyses, RF was run on each omic individually (Fig. 3A). In TCGA, taken together or separately, the three omics are highly predictive of cancer subtypes. Apart from netDx, all methods present MCC values larger or equal than 0.75 with a slight advantage for RF_Concat. Furthermore, it can be noticed that PIMKL is more closely related to the top performers than netDx. In line with the idea that SNR is high in oncology, these two observations match the profile obtained in the High_Main_MO scenario.

In RV144 and COVID19 on the other hand, higher heterogeneity across modalities can be noticed in terms of predictive power (Fig. 3A). In RV144, only transcriptomic data contain discriminant features, whereas all but metabolomic data have high MCC values in COVID19. In both cases, a higher SNR is found in the largest views. While these two datasets match the Main_MO_[12]largest_Omics scenarios, three notable differences exist between these simulations and the real datasets. (i) In RV144, BlockForest lags behind the other RF-based approaches and fails to detect the signal in transcriptomic data as illustrated by the relative uniform distribution of block weights (Supplementary Figures 8 and 9). (ii) In RV144 and COVID19, SIDA demonstrates the highest median MCC, whereas in Main_MO_[12]largest_Omics, RF-based approaches and DIABLO (in Main_MO_2largest_Omic) only outperform the others. (iii) PIMKL and netDx come second and third best in COVID19, whereas in Main_MO_2largest_Omics, they exhibited the lowest performances. Behind these apparent discrepancies, these results confirm the robustness of SIDA to the absence of main effect in smaller views that was already underlined in simulations. The elevated MCC values for PIMKL and netDx suggest on the other hand that COVID19 dataset contains a high fraction of discriminant features.

Finally, a careful observation of PIMKL and DIABLO’s weights (Supplementary Figures 6 and 7) reveals that they do not necessarily correlate well with single omic performances (Fig. 3A). The two methods show nevertheless a same hierarchy in their estimation of dataset importance.

## Discussion and conclusion

In this study we have benchmarked six methods representative of multi-omics data integration. The specificity of our work lies in its focus on classification and the use of a large number of simulation scenarios to understand the behavior of methods in a wide variety of settings. Non-integrative approaches were further included to characterize the conditions under which data integration offers a benefit. Interestingly, the underlying model seemed to be the main driver of the performances, especially on simulations. RF-based and to a lesser extent similarity-based methods showed homogeneous behavior in many scenarios and datasets. In term of performance, the latent variable models demonstrated their superiority when the main multi-omics effect was present in a subset of views (*i.e.* Main_MO_[12]Smallest_Omics, RV144 and COVID19). By contrast, RF-based approaches performed better (High_Main_MO) or equally well (TCGA) as the other methods, when the multi-omics effect was strong. Beyond this trend, it is important to note that on real data, integrative approaches perform better or equally well than non-integrative ones. This suggests that the benefits of data integration are more evident on real data than simulated. This in turn implies that, although useful for testing precise hypotheses, simulations do not fully recapitulate complex multi-omics signal.

At the method level, DIABLO outperformed the other methods on 6 out of 15 scenarios and stood out in the high dimensionality setting (px5) and when the signal was present in the smallest views only, pointing to its high ability to select discriminant features. The user should nevertheless keep in mind that weights are estimated on the training set, which can weaken the method’s accuracy when the multi-omics effect is absent from one or multiple views. SIDA showed increased performance in the presence of confounders or absence of signal in the smallest views, notably on RV144 and COVID19. By contrast, when the main effect was absent in the largest view, its MCC significantly decreased. When the fraction of discriminant features was high, PIMKL showed high performances in simulation and real-world datasets. Conversely, when the number of signal features was small, PIMKL and NetDx showed a reduced MCC. Even though the authors of EasyMKL, the method underlying PIMKL, observed that their method “obtains good results even with a 1000% of additional noise features,” the heterogeneous nature of omics data means that similarities computed on full datasets may not be very informative. To circumvent this limitation, (i) netDx includes a step of variable selection prior to network construction and (ii) the two methods recommend to integrate data at the pathway level.

In the light of these results, we therefore recommend that users conduct an initial step of analysis of variance on each modality to guide their choice toward the most appropriate methods. If the results reveal that the main effect is strong in all views, RF can be considered. If the number of samples is large and the main effect only present in the largest modalities, SIDA should be utilized. In other cases, DIABLO should be favored. When the focus is on deciphering biological mechanisms, PIMKL and netDx should be preferred.

In the present evaluation, methods were only assessed on a predictive criterion, ignoring thus the underlying biology. While significantly more difficult, it would be interesting in the future to evaluate these methods on the biological relevance of the selected features. Further extensions could also explore alternative models and parameters in the simulations (*e.g.* nonlinear latent factors, heterogeneity across modalities, noise distribution, etc.). One could argue that latent variable models were favored by MOFA whose underlying model also relies on linear latent factors.

**Key Points**

- Classification integrative methods have received little attention in the literature so far. In this work, six supervised methods spanning major families of integrative approaches are thoroughly evaluated.
- Non-integrative approaches (RF based) were further included to elucidate the conditions in which data integration provides a clear advantage.
- Latent variable models stood out both on simulations (DIABLO) and real data (SIDA).
- When the multi-omics effect was strong, RF-based approaches performed better (High_Main_MO scenario) or equally well (TCGA) as the other methods.
- PIMKL demonstrated increased performances when the fraction of discriminant features was high.

## Supplementary Material

Supplementary_bbae331

## Code availability

The code to reproduce the simulations and results is available on GitHub (https://github.com/bioaster/benchmark-integrative-methods); instructions to retrieve the real-world datasets are provided in the original studies [43, 44, 50].
