# Supplementary material for: Comparative analysis of integrative classification methods for multi-omics data
Source: Brief Bioinform. 2024 Jul 10;25(4):bbae331. doi: 10.1093/bib/bbae331 (PMC11234228; doi:10.1093/bib/bbae331)
Supplement: Supplementary_bbae331 [file supplementary_bbae331.pdf]

Supplementary Materials
Comparative analysis of integrative classification methods for multi-omics data

Alexei Novoloaca, Camilo Broc, Laurent Beloeil,
Wen-Han Yu and Jeremie Becker

1 Method Parametrization

SIDA: In the default version of the code, the sparsity parameter (lambda, *i.e.* variable selection) is optimized via a grid search, which is set to five values per omic when the number of omics (D) is larger than two. This amounts to 5^D values to be tested. The vector of lambda is generated by the sidatunerange function and in the case of our datasets varies between 0.45 and 0.75. This restricted range means that the method do not explore the full sparsity path and may lead to sub-optimal variable selection. To circumvent this limitation, the code was modified to expand the range to $[0, 1]$. The performances obtained on RV144 transcriptomic data (10,000 most variable genes) are shown in supplementary Figure 10 where the MCC is maximal for values between 0 and 0.4 and decreases for higher values. These observations prompted us to keep this modified range in the present study and led on average to a 0.1 increase in MCC (data not shown).

2 Tools and Packages Versions

The software and packages used in this study were up to date at the initiation of the benchmark (second half of 2021). Specifically, R 4.1.0 and Python 2.7.5 were utilized throughout this work, the versions of packages are provided in the main text (Table 1).

3 Hardware and Runtime

The analyses presented here were run on an AMD EPYC 7702 server with 80 cores operating at 3.4 GHz. The hardware configuration included 256 GB of memory, while the platform ran on CentOS Linux 7.9 x86_64. The average time of three simulations is provided hereafter (Table 1) for each method on the reference and px5 scenarios.

Table 1: Estimated runtime (minutes)

Method	Reference	px5
DIABLO	8.3	9.2
SIDA	29.3	127.5
PIMKL	0.4	10.7
netDx	2.3	2.4
Stacked Generalization	1	4.1
BlockForest	0.72	2.3
RF_Concat	0.2	1.1
RF_Max_Single_View	0.3	1.2

4 Supplementary Figures

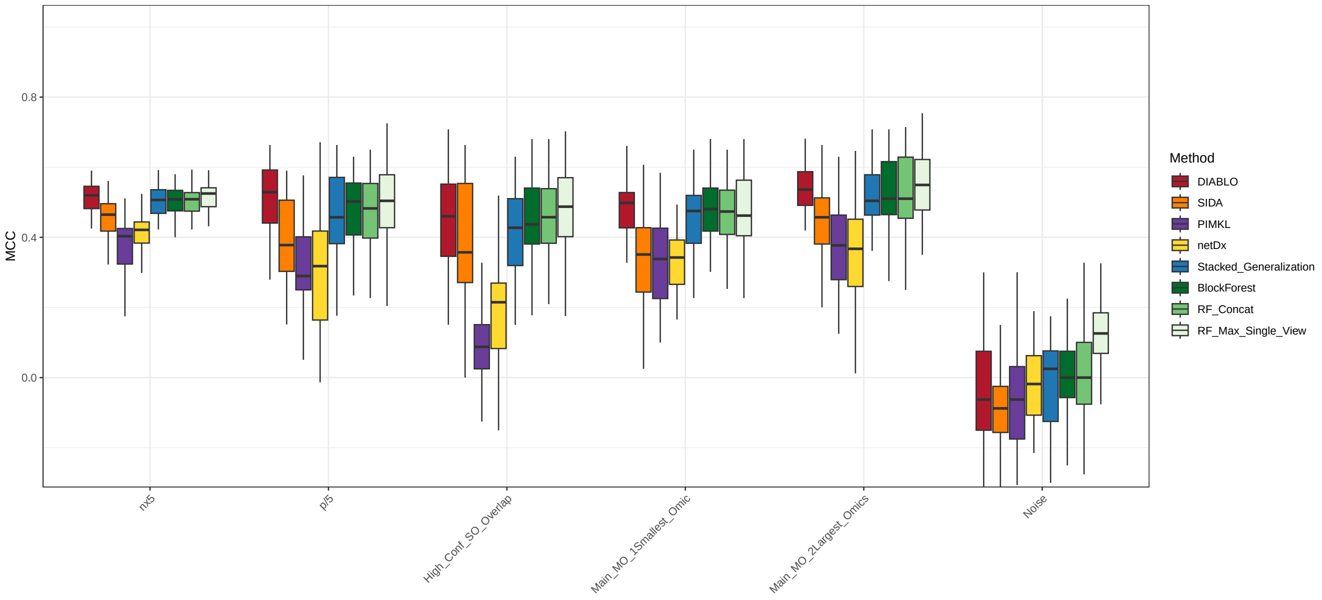

Figure 1: Method comparison on the 9 additional simulation scenarios. For each scenario, 40 repetitions were generated, on which, MCC was computed in 5-fold cross-validation.

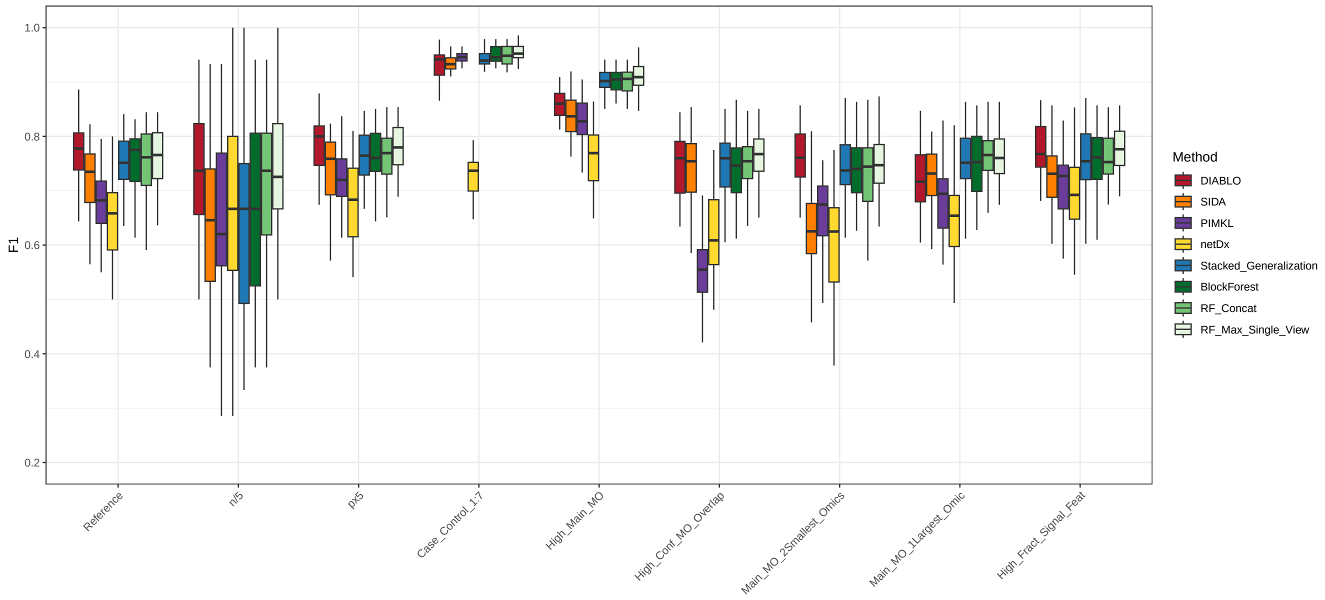

Figure 2: Method comparison on the main simulation scenarios. For each scenario, 40 repetitions were generated, on which, F1 was computed in 5-fold cross-validation.

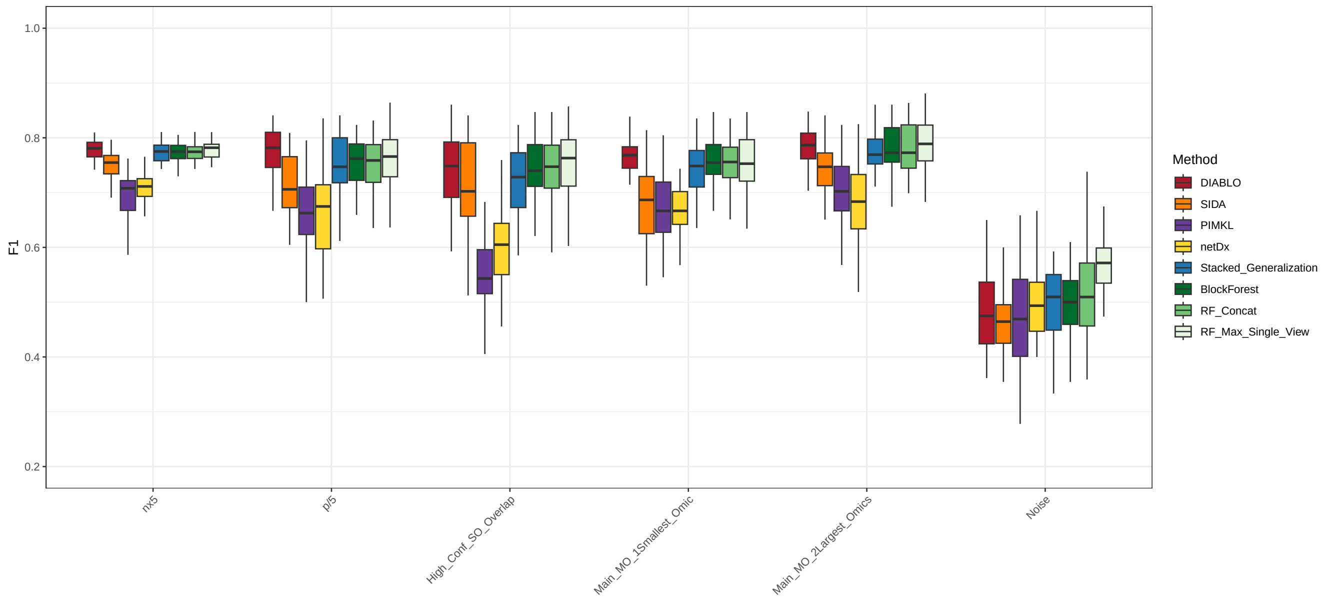

Figure 3: Method comparison on the supplementary simulation scenarios. For each scenario, 40 repetitions were generated, on which, F1 was computed in 5-fold cross-validation.

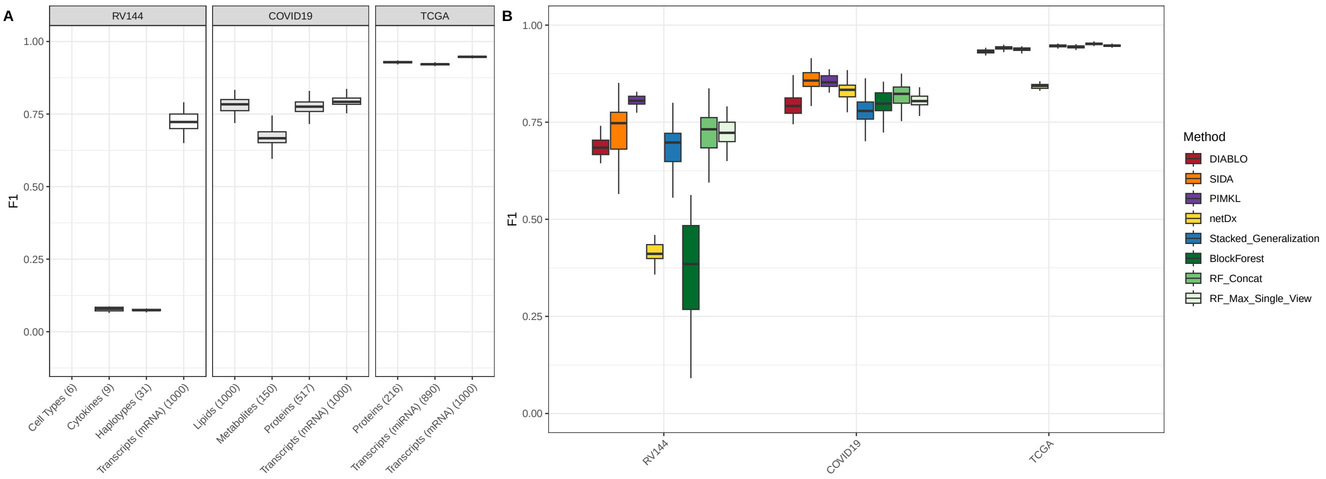

Figure 4: Method comparison on 3 real-world datasets. Prediction performance **(A)** on individual omic using Random Forest or **(B)** integrative methods. F1 was computed on 40 repetitions of 5-fold cross-validation.

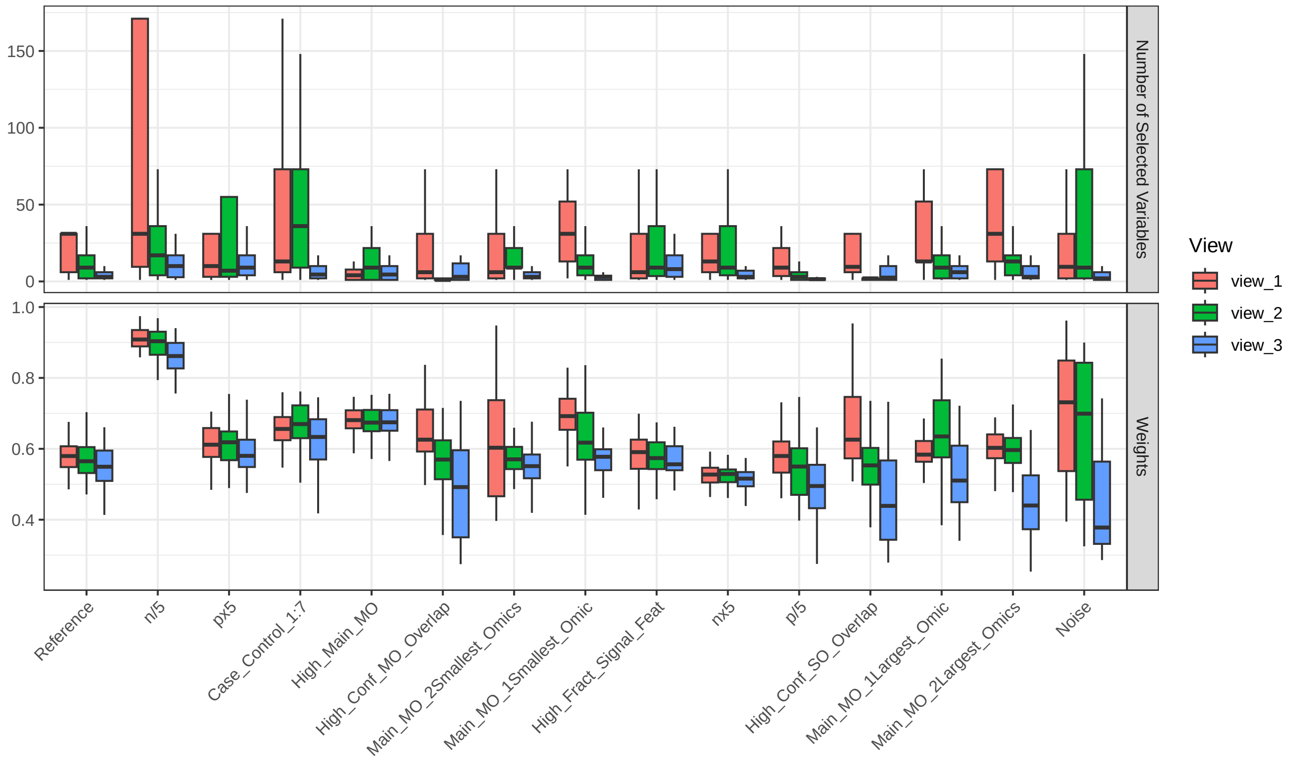

Figure 5: For each view, the number of variables selected (top) and weights estimated (bottom) by DIABLO on all 15 simulation scenarios. For each scenario, 40 repetitions were generated.

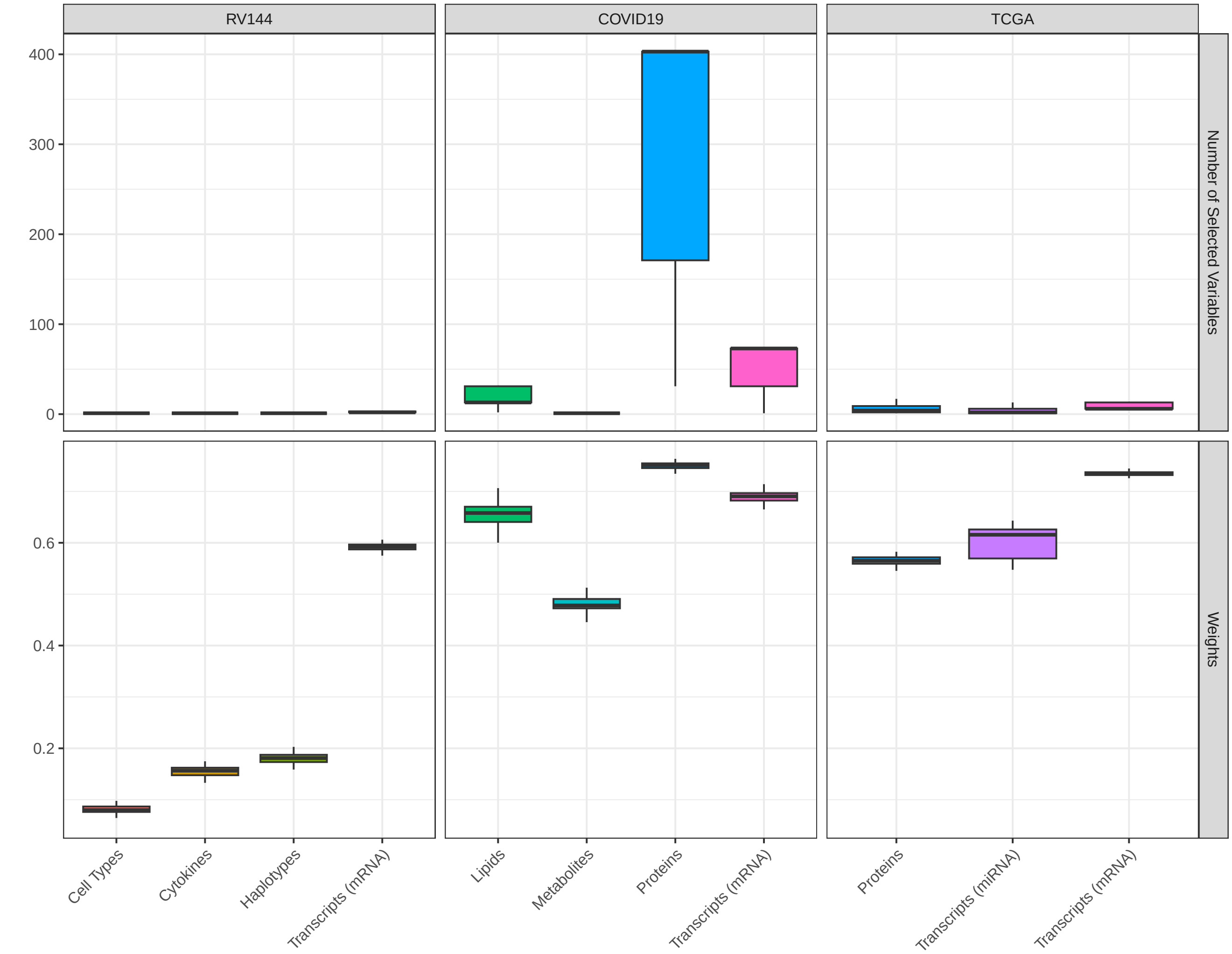

Figure 6: Number of variables selected (top) and weights estimated (bottom) by DIABLO on the 3 real datasets. For each scenario, 40 repetitions were generated.

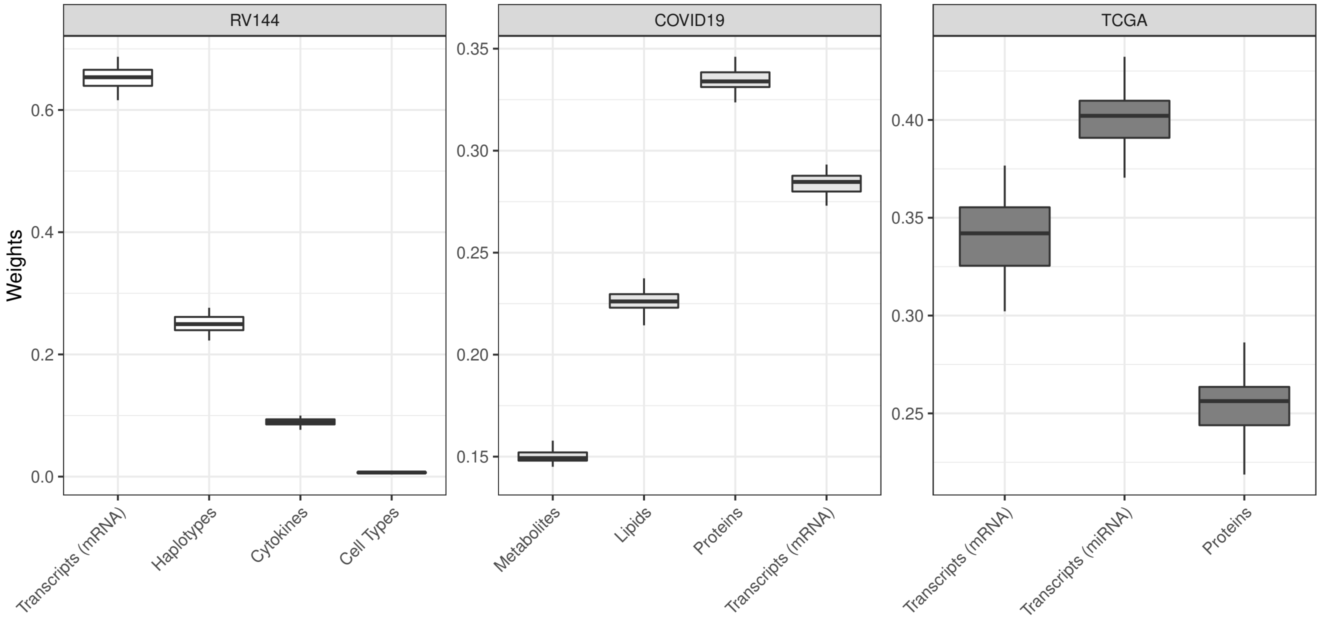

Figure 7: For each view, weights estimated by PIMKL on the 3 real-world datasets. For each dataset, 40 repetitions were generated.

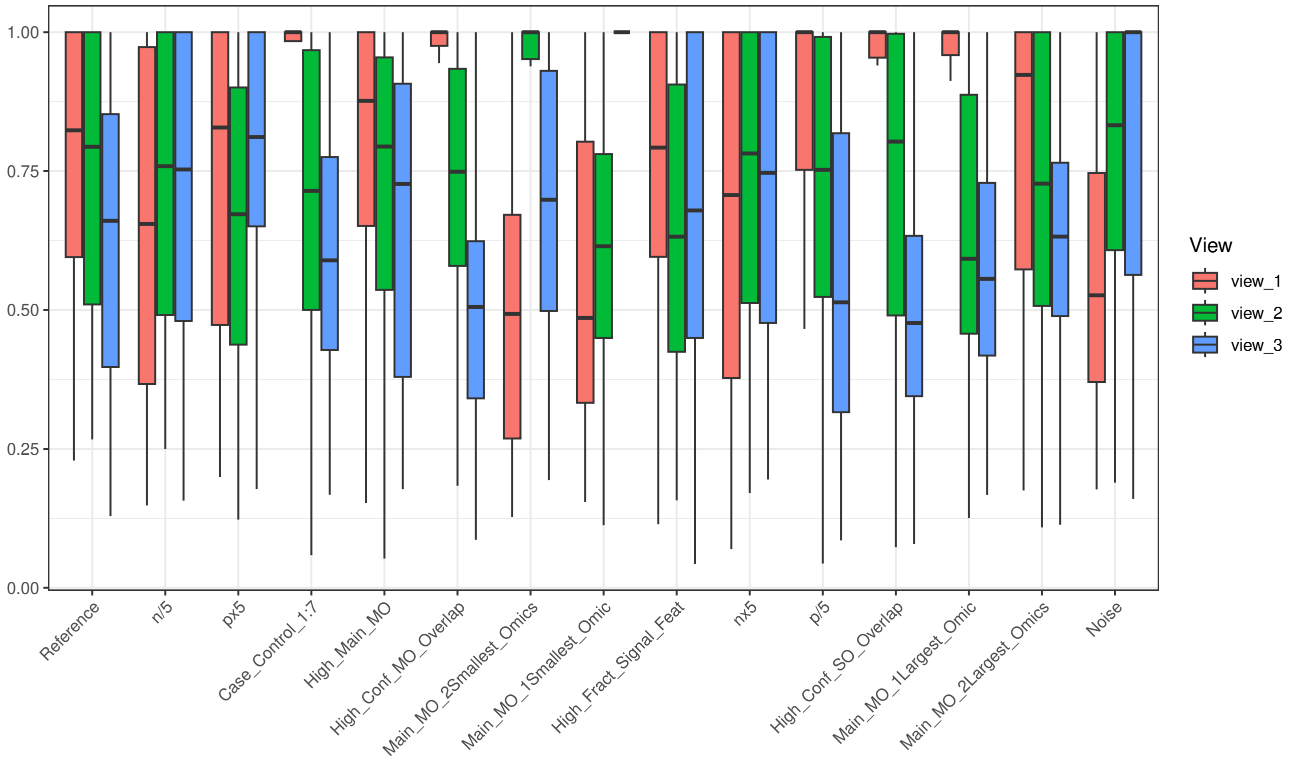

Figure 8: For each view, weights estimated by blockForest on all 15 simulation scenarios. For each scenario, 40 repetitions were generated.

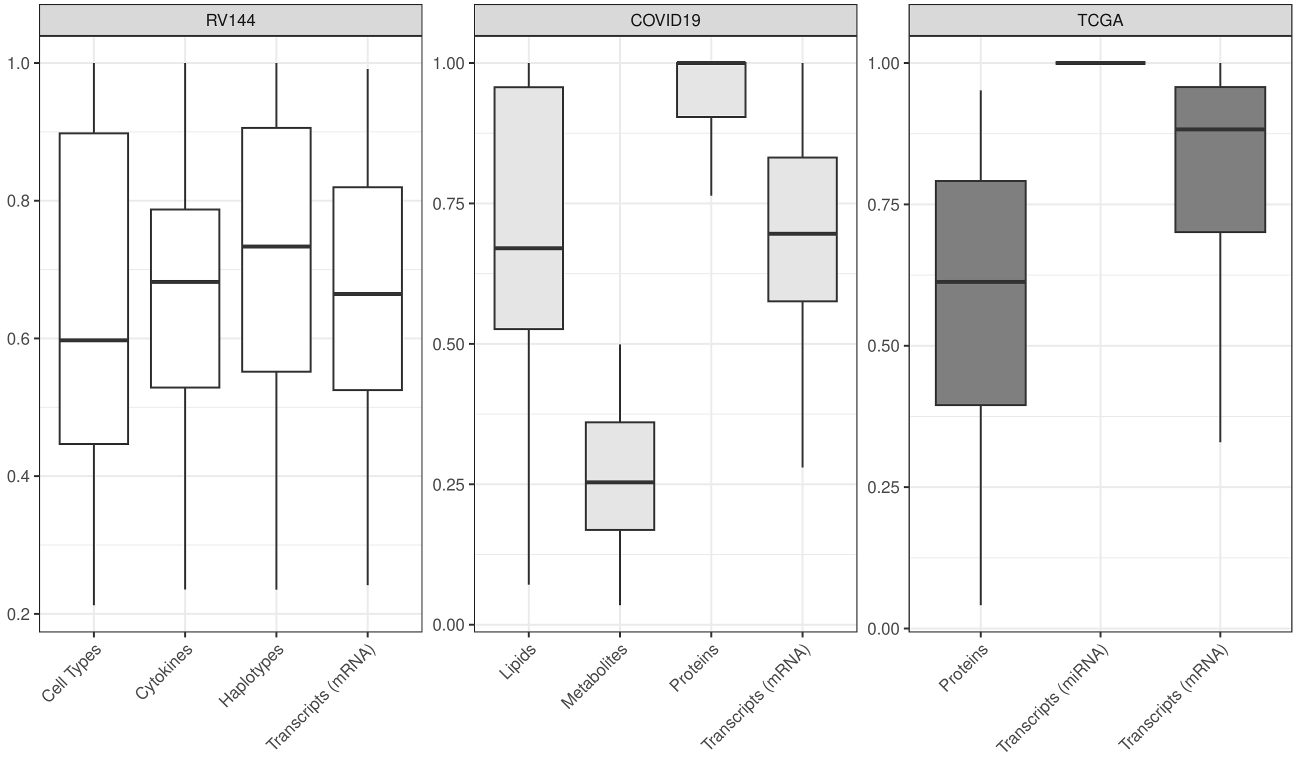

Figure 9: For each view, weights estimated by blockForest on the 3 real-world datasets. For each dataset, 40 repetitions were generated.

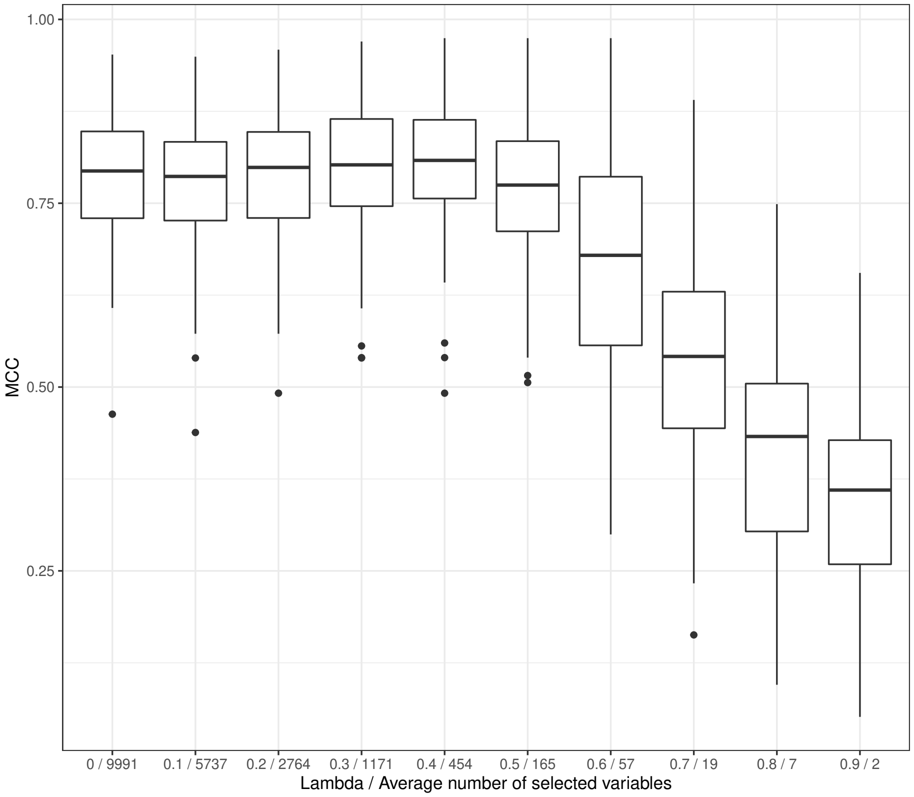

Figure 10: Evaluation of SIDA performances after increasing the default sparsity range. MCC was computed on 40 repetitions of 5-fold cross-validation.
